# Meetings of Societies

**Published:** 1933

**Authors:** 


### Meetings of Societies.

### Bristol Medico-Chirurgical Society.

SESSION 1932–33.

THE Annual General Meeting of the Society was held at the University on Wednesday, 11th October, 1933, at 8.30 p.m.

Dr. J. R. Charles was in the Chair until the arrival of the President, and seventy-eight members were present.

The Hon. Secretary read his report for the past year, and Mr. E. Watson-Williams read the annual report of the Hon. Editor of the *Journal*. These reports were adopted, and the Hon. Secretary and the Hon. Editor thanked for their work.

Dr. Carleton then proposed that Professor A. Rendle Short should be elected as President-Elect. This was seconded by Mr. Duncan Wood, and carried with acclamation.

Mr. Jackman proposed and Mr. E. Watson-Williams seconded that Mr. Shepherd should be re-elected as Hon. Secretary. This was carried. Dr. George Parker was unanimously re-elected as Hon. Medical Librarian on the proposition of Mr. E. Watson-Williams and seconded by Dr. Gee.

Dr. Frank Bodman proposed and Dr. Perry seconded that the following nine gentlemen be elected as members of the Committee :—

| | |
|---|---|
| Dr. Carleton. | Dr. A. L. Flemming. |
| Dr. Elwin Harris (jun.). | Dr. H. L. Fuller. |
| Mr. Jackman. | Dr. P. C. Martin. |
| Mr. Clifford Moore. | Dr. Vincent Wood. |
| Mr. Duncan Wood. | |

### BRISTOL MEDICO-CHIRURGICAL SOCIETY.

**INCOME AND EXPENDITURE ACCOUNT for the year ended 30th September, 1933.**

INCOME.

| | £ | s. | d. | £ | s. | d. |
|---|---|---|---|---|---|---|
| Members' Subscriptions .. | 330 | 4 | 6 | | | |
| Do., outstanding for 1932–1933 .. .. .. .. .. | 51 | 19 | 6 | | | |
| | | | | 382 | 4 | 0 |
| Interest on Bank Deposits .. .. | | | | 2 | 17 | 3 |
| Interest on £500 4 per cent. Consolidated Stock, less tax .. .. | | | | 15 | 0 | 0 |
| | | | | £400 | 1 | 3 |

EXPENDITURE.

| | £ | s. | d. | £ | s. | d. | £ | s. | d. |
|---|---|---|---|---|---|---|---|---|---|
| Grant to *The Bristol Medico-Chirurgical Journal* .. .. .. .. | | | | | | | 230 | 15 | 6 |
| Librarian—Honorarium .. .. .. | | | | | | | 15 | 0 | 0 |
| Miscellaneous Expenses— | | | | | | | | | |
| Goodway–Refreshments | | | | 16 | 0 | 0 | | | |
| Postages and Sundries | | | | 8 | 7 | 6 | | | |
| Printing and Stationery | | | | 12 | 15 | 0 | | | |
| Audit Fee .. .. .. .. | | | | 2 | 2 | 0 | | | |
| Lantern and Cinematograph at Meetings .. | | | | 6 | 6 | 0 | | | |
| W. Jewell—rehanging and reframing portraits | | | | 4 | 12 | 0 | | | |
| University Marshal .. | | | | 1 | 0 | 0 | | | |
| Lewis's Medical and Scientific Library— | | | | | | | | | |
| Subscription | 4 | 4 | 0 | | | | | | |
| Postages .. | 0 | 16 | 7 | | | | | | |
| | | | | 5 | 0 | 7 | | | |
| | | | | | | | 56 | 3 | 1 |
| | | | | | | | 301 | 18 | 7 |
| Balance, being excess of Income over Expenditure for the year, carried to Balance Sheet .. .. | | | | | | | 98 | 2 | 8 |
| | | | | | | | £400 | 1 | 3 |

**BALANCE SHEET as at 30th September, 1933.**

LIABILITIES AND CAPITAL.

| | £ | s. | d. | £ | s. | d. |
|---|---|---|---|---|---|---|
| Sundry Creditors .. .. | | | | 13 | 14 | 2 |
| Capital as at 30th Sept., 1932 .. .. .. .. .. | 680 | 1 | 6 | | | |
| *Add* Surplus on Income and Expenditure Account as above .. | 98 | 2 | 8 | | | |
| | | | | 778 | 4 | 2 |
| | | | | £791 | 18 | 4 |

ASSETS.

| | £ | s. | d. | £ | s. | d. |
|---|---|---|---|---|---|---|
| Cash at Bank— | | | | | | |
| Current Account .. .. | 138 | 16 | 1 | | | |
| Deposit Account .. .. | 163 | 13 | 9 | | | |
| | | | | 302 | 9 | 10 |
| £500 4 per cent. Consolidated Stock at cost .. .. .. .. .. .. | | | | 437 | 9 | 0 |
| Sundry Debtors—Members' Subscriptions for 1932–1933 outstanding .. | | | | 51 | 19 | 6 |
| | | | | £791 | 18 | 4 |

Audited and found correct,

H. E. KEELER,

*Chartered Accountant,*

*Auditor.*

16-18 Clare Street, Bristol 1,

10*th October*, 1933.

Dr. Phillips proposed and Dr. Zealand seconded that the following three members be elected to serve on the University Library Sub-Committee: Dr. G. Parker, Dr. Elwin Harris (sen.), Dr. Symes.

The Hon. Secretary then spoke of the Society's Annual Dinner, and a Dinner Committee was elected consisting of the President, Mr. Jackman and the Hon. Secretary.

The President, Mr. Hey Groves, then arrived, and after thanking Dr. Charles took the Chair. He proceeded at once to resign his office to his successor, Dr. Elwin Harris (sen.). A vote of thanks to the retiring President was proposed by Mr. Jackman and seconded by Dr. Elwin Harris (jun.). This was carried with acclamation.

The President then gave his inaugural address.

A vote of thanks to the President for his address was proposed by Mr. Carwardine and seconded by Mr. Chitty, and carried with acclamation.

The Hon. Treasurer then read his report for the past year. The report was adopted, and the Treasurer thanked for his work. Mr. Iles was re-elected Hon. Treasurer on the proposition of Mr. Wright, seconded by Mr. Clifford Moore.

---

The second meeting of the session was held at the University on Wednesday, 8th November, 1933, at 8.30 p.m.

Dr. ELWIN HARRIS was in the Chair, and fifty-eight members were present.

Dr. A. L. TAYLOR and Dr. T. B. WANSBROUGH showed pathological specimens and skiagrams respectively.

The minutes of the Annual Meeting were read and confirmed.

Professor R. S. S. STATHAM then read a paper on "**Vaginal Discharge.**"

A discussion then followed, in which Mr. SMYTHE, Mr. SHEPHERD, Mr. WALTERS and the PRESIDENT spoke. Professor STATHAM replied, and at his request Dr. A. D. FRASER spoke on the pathology of the problem.

The third meeting of the session was held at the University on Wednesday, 13th December, 1933, at 8.30 p.m.

Dr. ELWIN HARRIS was in the Chair, and forty-nine members were present.

Dr. BERGIN and Dr. FRASER showed X-rays and pathological specimens respectively.

Mr. E. WATSON-WILLIAMS showed a rhinolith removed at operation.

The question of the times of meetings was again read, and after the Hon. Secretary had explained the situation and several members had spoken in favour of continuing the later hour, the PRESIDENT made a proposition from the Chair that the meetings should continue to be held at 8.30 p.m. This was carried unanimously.

The PRESIDENT then called on Professor J. A. NIXON, who read a paper entitled "**The Recognition of Inherited Syphilis.**" A long and interesting discussion followed, in which the PRESIDENT, Dr. RICHARD CLARKE, Dr. CHARLES, Dr. BIRRELL, Dr. GEE, Dr. FRANK BODMAN, Dr. ALEXANDER, Dr. GARDEN, Mr. FITZGIBBON, Mr. SHEPHERD, and Mr. E. WATSON-WILLIAMS spoke, and Professor NIXON replied.

